# Mechanism of Anti-Diabetic Activity from Sweet Potato (*Ipomoea batatas*): A Systematic Review

**DOI:** 10.3390/foods12142810

**Published:** 2023-07-24

**Authors:** Cokorda Istri Sri Arisanti, I. Made Agus Gelgel Wirasuta, Ida Musfiroh, Emmy Hainida Khairul Ikram, Muchtaridi Muchtaridi

**Affiliations:** 1 Department of Pharmaceutical Analysis and Medicinal Chemistry, Faculty of Pharmacy, Universitas Padjadjaran, Sumedang 45363, Indonesia; cokorda22001@mail.unpad.ac.id (C.I.S.A.); ida.musfiroh@unpad.ac.id (I.M.); 2 Pharmacy Department, Faculty of Mathematic and Natural Science, Udayana University, Kampus Bukit Jimbaran, Bali 80361, Indonesia; gelgel.wirasuta@unud.ac.id; 3 Centre for Dietetics Studies, Faculty of Health Sciences, Universiti Teknologi MARA Cawangan Selangor, Kampus Puncak Alam, Bandar Puncak Alam 42300, Malaysia; emmy4546@uitm.edu.my; 4 Integrated Nutrition Science and Therapy Research Group (INSPIRE), Faculty of Health Sciences, Universiti Teknologi MARA Cawangan Selangor, Kampus Puncak Alam, Bandar Puncak Alam 42300, Malaysia; 5 Research Collaboration Center for Radiopharmaceuticals Theranostic, National Research and Innovation Agency (BRIN), Sumedang 45363, Indonesia

**Keywords:** *Ipomoea batatas*, flavonoid, phenolic acid, anthocyanins, type 2 diabetes, mechanism of action

## Abstract

This study aims to provide an overview of the compounds found in sweet potato (*Ipomoea batatas*) that contribute to its anti-diabetic activity and the mechanisms by which they act. A comprehensive literature search was conducted using electronic databases, such as PubMed, Scopus, and Science Direct, with specific search terms and Boolean operators. A total of 269 articles were initially retrieved, but after applying inclusion and exclusion criteria only 28 articles were selected for further review. Among the findings, four varieties of sweet potato were identified as having potential anti-diabetic properties. Phenolic acids, flavonols, flavanones, and anthocyanidins are responsible for the anti-diabetic activity of sweet potatoes. The anti-diabetic mechanism of sweet potatoes was determined using a combination of components with multi-target actions. The results of these studies provide evidence that *Ipomoea batatas* is effective in the treatment of type 2 diabetes.

## 1. Introduction

The prevalence of diabetes in 2021 was 537 million people [1]. This number is anticipated to rise by 10.2% by 2030 and 10.9% by 2045 [2]. Diabetes mellitus (DM) and its complications were responsible for 12.2% of fatalities worldwide in the age group from 20 to 79 years old in 2021 [1].

The most prevalent form of diabetes is type 2, which is characterized by impaired hepatic glucose metabolism, reduced pancreatic beta cell function, and peripheral insulin resistance [3]. The American Association of Clinical Endocrinologists (AACE) recommend α-glucosidase inhibitors as the first-line therapy because they are safe, effective, have a low incidence of hypoglycemia, and have tolerance in the cardiovascular system [4]; however, it has been claimed that this medication produces undesirable side effects [5]. Therefore, an investigation of natural ingredients that are both effective and safe has the potential to mitigate the risk of type 2 diabetes and its associated complications.

Sweet potato (*Ipomoea batatas*) is the sixth most grown food worldwide [6]. Its leaves are renowned for their antioxidant capabilities, surpassing those of ascorbic acid, tea, and grape seed polyphenols by a factor of 3.1, 5.9, and 9.6, respectively [7]. Remarkably, the leaf parts of 40 sweet potato cultivars contain a significant amount of polyphenols ranging from 7.39 to 14.66 g/100 g dry weight (DW) [8]. Within sweet potato leaves, phenolic acids, anthocyanins, and caffeoylquinic acid derivatives were identified as contributors to the observed hypoglycemic effects [9]. Sweet potato leaf ethanol extract obtained from Aan village, Klungkung, Bali consists of diverse flavonoids, such as anthocyanins, flavonols, and flavones, whose concentrations in the extract exhibited a linear correlation with the decrease in blood glucose and malondialdehyde levels [10]. Additionally, the type and concentration of phytochemicals found in sweet potatoes affect their anti-diabetic action [11]. Despite numerous studies investigating the anti-diabetic effects and mechanisms of *Ipomoea batatas*, comprehensive documentation is lacking. Therefore, this systematic review aims to provide an overview of the compounds responsible for the anti-diabetic activity and to elucidate their mechanisms of action. This review will function as a comprehensive database, aiding other researchers in identifying the subsequent steps for the development of *Ipomoea batatas*-based products.

## 2. Materials and Methods

### 2.1. Literature Search

The Preferred Reporting Items for Systematic Reviews (PRISMA) served as the foundation for the search approach [12]. The literature search in this systematic review aimed to find relevant articles about the potential of *Ipomoea batatas* for type 2 diabetes treatment. We comprehensively selected electronic databases such as PubMed, Scopus, and Science Direct. Boolean operators were used to conduct the literature search [13]. The keys included (1) *Ipomoea batatas* OR sweet potato AND (2) diabetic OR type 2 diabetes.

### 2.2. Inclusion Criteria

For an article to be included in this study the anti-diabetic potential, chemical components, and mode of action of *Ipomoea batatas* needed to be covered in research articles based on in vitro and in vivo experiments. The selected article had to be written in English and should have evaluated at least the following: (1) *Ipomoea batatas*, (2) chemical components, (3) anti-diabetic effects, and (4) mechanisms of action involved.

### 2.3. Exclusion Criteria

Articles not included in the systematic review were in the form of proceedings; theses; dissertations; review articles; articles not written in English; articles with titles, abstracts, and keywords that did not meet the inclusion criteria; and articles that focused on other diseases.

### 2.4. Study Selection

The full text of the relevant published article was then reviewed. The articles that were chosen to be included in this study were compiled using Mendeley, a reference manager.

### 2.5. Data Extraction and Management

The articles that met the inclusion criteria were then analyzed, and the data collected included (1) type/cultivar, (2) material used, (3) detected phytochemical compound, (4) predicted bioactive compound, (5) type of study, (6) dose, (7) action and mechanism of anti-diabetic activity of *Ipomoea batatas*.

## 3. Results

### The Literature Search

**Figure 1:**
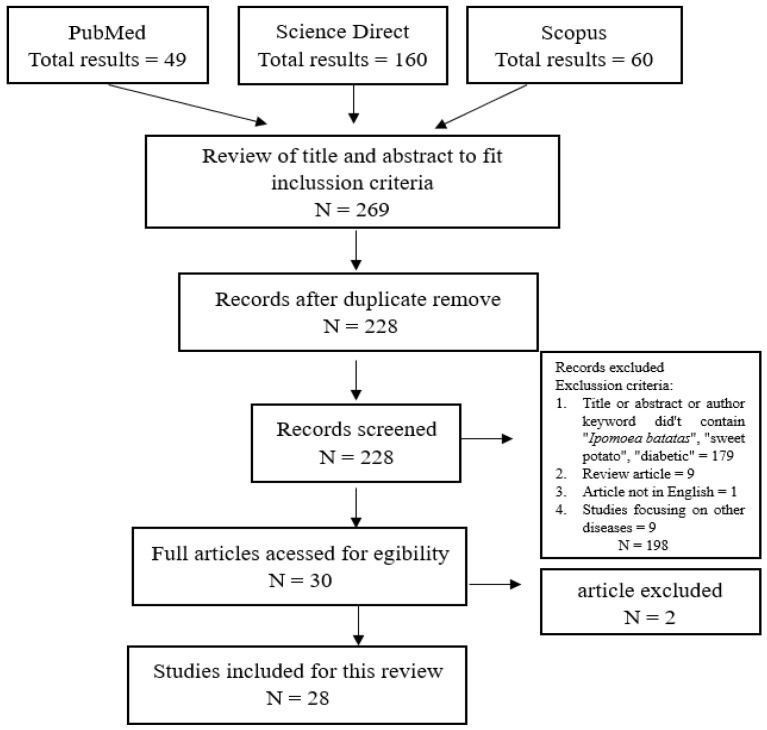
Flow chart showing the literature search.

A literature search was able to identify 269 articles relevant to the topic. After duplication detection, 41 papers were deleted. Based on the title, abstract, keywords, and inclusion criteria mentioned above, an additional 198 articles were excluded. Two reports could not be accessed in the full paper version, so finally 28 papers were discussed in depth in this review. A flow diagram summarizing the filtering, identification, and reasons for exclusion is shown in Figure 1. Data extraction was performed based on the selection of completed articles, as shown in Table 1.

## 4. Discussion

### 4.1. Varieties of Ipomoea Batatas Developed for Type 2 Diabetes

Sweet potatoes are distinguished by their color, width, thickness, shape of the leaves, size, and color of the skin and flesh of the tubers [40]. Research on the anti-diabetic activity includes white [14,15,16,23,24,28,30,31,32,34], purple [20,22,33,35,37,39], orange [29,38], and Japanese green sweet potatoes [25]. Understanding of the *Ipomoea batatas* varieties that are proven to exhibit anti-diabetic activity will facilitate the identification and isolation of specific bioactive components that can serve as starting molecules or models for creating a novel synthetic medicine [41,42].

### 4.2. Types and Concentrations of Phytochemicals Contained in Ipomoea batatas Which Have Anti-Diabetic Effects

The leaves of white sweet potato have a total polyphenol concentration of 6.4 g/100 g, which is greater than that of the orange varieties as well as Japanese green sweet potatoes [16,25,38]. The plant parts used also have an impact on the variation in polyphenol concentration. The total polyphenols in the leaves are more significant when compared to the tuber [43]. Green leaves have higher total phenolics than green or purple leaves [44]. Different maturity stages of sweet potato plants exhibit a significant amount of variation in flavonols and phenolic acids of the sweet potato leaves. The quantity of bioactive compounds rises as the plant ages [45]. Anthocyanin concentrations are more significant in purple than orange tuber sweet potatoes. The concentration of phenolic acids in purple tubers is ten times greater than that in orange and white sweet potatoes [46].

### 4.3. Mechanism of Action Chemical Components in Ipomoea batatas for Anti-Diabetic Effects

#### 4.3.1. Protects the Integrity of Islet Structures and Modulates Pancreatic β Cell Function

β-pancreatic cells are responsible for insulin secretion. Therefore, maintaining the islet structure of pancreatic β cells is essential for treating diabetes. The results of the pancreatic histopathological analysis showed that the administration of white sweet potato ethanol extract at doses of 80 and 150 mg per kg BW of mice for four weeks could improve the islet structure by enlarging the islet area and inhibiting apoptosis of β-pancreatic cells [47,48]. In addition, administering purple sweet potato extract containing anthocyanins and protein at a dose of 200 mg/kg body weight reduced oxidative stress and pancreatic damage in diabetic mice [20]. However, a larger dose of cloned B 0059-3 sweet potato extract obtained from Bandungan, West Java, Indonesia was required to protect β-pancreatic cells [19]. The ability to protect and modulate the function of pancreatic β polyphenols contained in ethanol extracts of white and purple sweet potato is more significant than resveratrol and polyphenols contained in Ginger (*Zingiber officinale*) rhizome [49,50]. The administration of polyphenol or protein-bound anthocyanins and free anthocyanins induced the expression of AMP-activated protein kinase (AMPK) in the liver, significantly increased levels of glucose transporter type 2 (GLUT2), glucokinase protein (GK), and insulin receptor α (INSR) [20,51].

#### 4.3.2. Increased Insulin Secretion and Improved Insulin Sensitivity

In vivo studies have demonstrated that the administration of Caiapo, glycoprotein acid, and 3,4,5-tricaffeoylquinic results in an increased insulin sensitivity [15,24,25,52]. The effectiveness of Caiapo as an antidiabetic was proven by conducting clinical trials on 30 patients given Caiapo 4 g/day orally, once a day, in the morning before meals. Caiapo administration led to a significant reduction in HbA1c compared to the placebo group after 2 and 3 months of the administration. In addition, from the study’s results, it was found that the administration of Caiapo caused the average fasting blood glucose level to reach 126 mg/dl, weight loss, and a significant decrease in postprandial glucose levels and cholesterol [14]. The caffeoylquinic derivative significantly increased glucagon-like peptide-1 (GLP-1) secretion [25,53] and glycoprotein acid increased modulation of insulin sensitivity (adiponectin) [28,54]. Similar results were obtained from the administration of polyphenols, such as phenolic acids and flavonoids, from the sweet potato leaf extract, with an improved insulin sensitivity through activation of insulin signaling in the skeletal muscles [23]. Flavonoids, such as methyl decanoate, have the potential to increase insulin sensitivity in skeletal muscles [22,55,56,57]. The increased insulin sensitivity is due to Akt phosphorylation, thereby activating insulin signals in the skeletal muscles of phosphatidylinositol 3-kinase/protein kinase B/glucose transporter 4 (PI3K/AKT/GLUT-4) and liver (PI3K/AKT/GSK-3β) [23,31,47,58,59,60]. 

#### 4.3.3. Regulation of Carbohydrate Metabolism

Ethyl caffeate has the ability of α-glucosidase inhibition with an IC_50_ value 6.77 times lower than acarbose. Flavonoids, such as kaempferol, quercetin, hyperoside, isoquercitrin, and rutin, also showed a stronger inhibition of α-glucosidase compared to acarbose [16]. Phenethyl cinnamates, 3,4,5-tricaffeoylquinic acid, quercetin-3-O-glucosidase, and 7-hydroxy-5-methoxy coumarin also showed excellent α-glucosidase inhibitory activity, where the IC_50_ values were much lower than that of the positive control acarbose. The increasing number of caffeoyl groups bound to quinic acid and methoxylation in flavonol compounds led to increased inhibition of α-glucosidase [29,61]. The inhibitory ability of α-glucosidase by phenolic acids and flavonoids is possible through binding enzyme surface amino acid residues, thereby altering the conformation of α-glucosidase, distorting the active site, and decreasing enzyme activity [53]. Ethyl caffeate, quercetin, hesperetin, luteolin, rutin, catechins, and cyanidin-3-glucoside also have an α-amylase inhibitory action [16,62]. Anthocyanins and protein-bound anthocyanins were able to control the expression of genes essential in glycolysis, such as phosphofructokinase (PFK) and pyruvate kinase (PK), and suppress the expression of gluconeogenic genes glucose-6-phosphatase (G6Pase) and phosphoenolpyruvate carboxykinase (PEPCK) [20].

#### 4.3.4. Suppression of Glucose Production in the Liver

Glycoproteins may play a role in suppressing gluconeogenesis [31,63,64]. The administration of acetylated anthocyanins cyanidin, 3-caffeoyl-p-hydroxybenzolsophoroside-5-glucoside, peonidin, and 3-caffeoyl sophoroside-5-glucoside have been shown to decrease glucose production in HepG2 cells. However, only cyanidin reduced the fasting blood glucose levels to 186–205 mg/dL after 1 and 2 h of in vivo administration [39].

#### 4.3.5. Inhibition of Glucose Transport in the Intestine and Increased Uptake of Tissue Glucose

The administration of hexane and a water fraction of purple sweet potato leaf methanol extract increased the glucose uptake in 3T3-L1 adipocyte tissue and rat hepatocytes. Flavonoids, such as quercetin, have a more remarkable glucose uptake ability than other components such as 3-O-β-D-sophoroside, benzyl β-D-glucoside, and 4-hydroxy-3-methoxy benzaldehyde. The ability of some of these active compounds in glucose uptake in adipocyte tissue is most likely through the activation of GLUT4 and regulation of the phosphatidylinositol 3-kinase (PI3K)/AKT pathway [22,65]. However, the administration of 5% white sweet potato powdered leaves increased the expression of p-IR, p-AKT, and M-GLUT4, but had no significant effect on the PI3K/AKT pathway [23].

#### 4.3.6. Repair of Insulin Signals and Glycogen Synthesis

There was an increase in mRNA insulin receptor (IR) expression, insulin receptor substrate 2 (IRS-2), PI3K, and AKT genes, and a decrease in glycogen synthase kinase-3β (GSK-3β) expression with white sweet potato extract administration [16]. This proved that these extracts promote liver glycogen synthesis by activating the insulin-mediated PI3K/AKT/GSK-3β signaling pathway [47,66]. Moreover, the administration of ethyl acetate fraction from white sweet potato ethanol extract and flavonoids contained in the water fraction of the extract was able to activate GLUT4 and regulate the phosphatidylinositol 3-kinase (PI3K)/AKT pathway [36].

#### 4.3.7. Inhibition of Inflammatory Pathways

The commercial administration of anthocyanins from purple sweet potato decreased the expression of cyclooxygenase-2, tumor necrosis factor-α, interleukin (IL)-1β, and IL-6. Anthocyanins can inhibit the phosphorylation induction/activation of extracellular signal-regulated kinase (ERK), c-Jun N-terminal kinase (JNK), and p38 mitogen-activated protein kinase (MAPK) [67]. The administration of 5 g/kg BW/day Caiapo significantly decreased p38 MAPKs and TNF-α production in diabetic rats. These findings imply that the inhibition of oxidative stress and the creation of pro-inflammatory cytokines, followed by an increase in the pancreatic cell mass, are what cause the hypoglycemic effects of *Ipomoea batatas* [27].

## 5. Conclusions and Perspective

Sweet potatoes that have the potential to be anti-diabetic include white, purple, orange, and Japanese green sweet potatoes. Phenolic acids, flavonols, flavanones, and anthocyanidins are responsible for the anti-diabetic activity of sweet potatoes. The anti-diabetic mechanism of sweet potatoes is determined by a combination of components with multi-target actions.

Given the increasing prevalence of diabetes, it is crucial to conduct research on the utilization of unstudied sweet potato varieties and cultivars. Additionally, implementing quality control measures to ensure product uniformity during production is imperative for medicinal purposes. A comprehensive approach must be taken to ensure consistency in the quality, efficacy, and safety of sweet potatoes as an anti-diabetic treatment. Although numerous studies have described the benefits of the bioactive compounds of *Ipomoea batatas* as anti-diabetic agents, there are still some limitations. The type and concentration of the bioactive compounds of *Ipomoea batatas* are influenced by many factors such as genetics, the time of harvest, the post-harvesting process, and the extraction process. Standardization and quality control are necessary to guarantee the consistency of the type and amount of bioactive components responsible for the anti-diabetic effect. Standardized, validated, and characterized herbal drugs, along with their identified biochemical compounds, can be used in clinical trials and, subsequently, could contribute to advancements in the pharmaceutical industry. The quality marker (Q-marker) concept emphasizes the relationship between chemical components, manufacturing processes, and the efficacy and safety of herbal medicines. Similarly, there needs to be an accurate determination of the pharmacokinetics and dynamics of polyphenols contained in sweet potatoes. Therefore, further studies are required to determine the Q-marker for quality control of *Ipomoea batatas* as an anti-diabetic agent, as well as to investigate the bioavailability of its active components.

## Figures and Tables

**Table 1:** Study of cultivar variation, chemical components, anti-diabetic activity, and mechanism of action of *Ipomoea batatas*.

Type/Cultivar	Part of Material	Detected Phytochemical Compound	Predicted Bioactive Compound	Type of Study	Dosage	Action and Mechanism	Refs.
White sweet potatoes	Powdered white sweet potatoes (Caiapo)	-	Acidic glycoprotein	In vivo	4 g/day for 12 weeks.	Decreased HbA1c Decreased fasting blood glucose Decreased two-hour glucose level Lowering mean cholesterol Decreased body weight	[14]
White sweet potato	Lyophilized powder of skin or flesh and combination skin and flesh aqueous extracts	85% Ethanolic extract 15% TCA supernatant Active fraction Ultrasonic fraction	Acidic glycoprotein	In vivo	20–2000 mg/kg BW/day for 3 weeks	Increased insulin activity Ultrasonic fraction decreased body weight and blood glucose, and increased blood insulin level compared to the control group	[15]
White sweet potato cultivar Simon No. 1	Freeze-dried 70% leave ethanolic extract Sweet potato leave phenolic acid (SPLPA) was purified using dynamic adsorption and desorption on AB-8 resin Sweet potato leave flavonoid (SPLF) was purified using liquid–liquid extraction	The phenolic acids were 8 isomeric caffeoylquinic acids, esculin, protocatechualdehyde, CA, 7- hydroxycoumarin and ethyl caffeate The flavonoids were rutin, hyperoside, isoquercitrin, astragalin, quercetin, kaempferol, diosmetin, jaceosidin, chrysin, and pectolinarigenin	Protocatechualdehyde Ethyl caffeate Quercetin	In vitro	(0.25–100 µg)	Antioxidant activity (DPPH, ABTS, and FRAP assay) α-glucosidase inhibition α-amylase inhibition	[16]
Sweet potatoes were collected from the Hebei province district in October	40 to 90% ethanolic viscous leaves extract	Flavonoids	-	In vivo	50–150 mg/kg BW for 28 days	Decreased in the concentration of fasting blood glucose (FBG), total cholesterol (TC), and triglyceride (TG) in diabetes mellitus mice Increased body weight (BW) and serum high-density lipoprotein cholesterol (HDL-c) level	[17]
Sweet potatoes were obtained by a local farmer (Hebei province) in autumn	Flavone extracts	Flavones	-	In vivo	25–100 mg/kg BW for 2 weeks	Decreased in the concentration of plasma triglyceride (TG), plasma cholesterol (TC), and weight in NIDDM rats Decreased fasting plasma insulin level, blood glucose (FBG) level, low-density lipoprotein cholesterol (LDL-C), and malondialdehyde (MDA) levels, and significantly increased the insulin sensitive index (ISI) and superoxide dismutase (SOD) level in NIDDM rats	[18]
Sweet potato (family of clones B 0059- 3) were harvested in July from the Bandungan, Central Java Indonesia.	Evaporated petroleum ether leave extract	Flavonoids Phenolic content	-	In vivo	0.25–0.8, 2.5 g/kg BW for 14 days	Lowest fasting blood glucose among treatment groups Normalizing functional beta cells	[19]
Purple sweet potatoes (Cultivar Eshu No.12) from the Institute of Food Crops, Hubei Academy of Agricultural Sciences (Wuhan, China)	Protein-bound anthocyanin compounds (p-BAC-PSP) Free anthocyanin compounds (FAC-PSP)	Total anthocyanin content and protein	13 different anthocyanins (cyanidin-3-sophoroside-5- glucoside, peonidin-3-sophoroside-5-glucoside) 17 protein groups	In vivo	p-BAC-PSP (500 mg/kg BW) FAC-PSP (200 mg/kg BW) with total anthocyanin content in FAC-PSP = 40.74 ± 2.88 mgC3G/g	Improvement of glucose tolerance and lipid metabolism Decreased oxidative stress and liver damage of diabetic mice Induced the expression of AMP-activated protein kinase (AMPK) in the liver. With p-BAC-PSP or FAC-PSP treatment, glucose transporter type 2 (GLUT2), the protein levels of glucokinase (GK), and insulin receptor α (INSR) Up-regulated glycolysis key genes, phosphofructokinase (PFK), pyruvate kinase (PK) Down-regulated gluconeogenic genes, glucose-6-phosphatase (G6Pase), and phosphoenolpyruvate carboxykinase (PEPCK)	[20]
The sweet potato was purchased from the local market of Faisalabad (Pakistan).	Evaporated methanolic extract	-	Glycoprotein Anthocyanins Alkaloids Flavonoids	In vivo	4 g/kg BW/day for 14 days	Decreased blood glucose level, protein glycation level, total cholesterol, triglycerides, and low-density lipoprotein (LDL)-cholesterol. Increased in high-density lipoprotein (HDL)-cholesterol level Beneficial effects on total protein concentration, albumin, globulin, and liver enzymes (serum glutamic oxaloacetic transaminase (SGOT), and serum glutamic pyruvic transaminase (SGPT))	[21]
Purple sweet potato leaves were collected in Luzhu District, Taoyuan City, Taiwan	Crude extracts, including n-hexane- (IBH), 95% MeOH- (IBM), n-BuOH- (IBB), and H_2_O-soluble (IBW) fractions	Twenty-four pure compounds	Quercetin 3-O-β-D-sophoroside Quercetin Benzyl β-d-glucoside, 4-hydroxy-3 methoxybenzaldehyd Methyl decanoate	In vitro	Crude extract (0.1 mg/mL) pure compounds (0.01 mg/mL) were	Increased glucose uptake, most likely via activation of Glut4 and regulation of the PI3K/AKT pathway	[22]
White sweet potato Tainung No. 10	Lyophilized powder of leave and tuber	-	Flavonoids, terpenoids, tannins, saponins, glycosides, alkaloids, steroids, and phenolic acids in tuber	In vivo	Powdered leaf: 5–50 mg/kg BW Powdered tuber: 100–300 mg/kg BW	Lowered plasma glucose, insulin, glucose area under the curve (AUC), homeostatic model assessment of insulin resistance (HOMA-IR), alanine transaminase, triglyceride, and tumor necrosis factor alpha levels. Restoration of the Langerhans’s areas Increased expression of insulin-signaling pathway-related proteins, phosphorylated insulin receptor and protein kinase B, and membrane glucose transporter 4 Inducing pancreatic islet regeneration and insulin resistance amelioration	[23]
White sweet potato	Powdered white sweet potatoes (Caiapo)	-	-	In vivo	2–4 g/d for 6 weeks	Increased insulin sensitivity No significant changes were seen in any of the parameters related to insulin dynamics: insulin secretion (from C-peptide), distribution, clearance, and hepatic extraction remained virtually the same after the treatment Improved metabolic control in type 2 diabetic patients by decreasing insulin resistance without affecting body weight, glucose effectiveness, or insulin dynamics	[24]
Leaves and stems of ‘Suioh’ which was harvested in the summer of 2009 in Kumamoto prefecture, Japan	Lyophilised powder of 60% ethanolic extract	CQA derivatives Mono-CQAs Di-CQAs Tri-CQAs	Total polyphenols CQA derivatives g Mono-CQAs In-CQAs Tri-CQAs	In vitro and in vivo	2 g/kg BW/day for 5 weeks	Lowered glycemia in type 2 diabetic mice. Sweet potato extract and CQA derivatives significantly enhanced glucagon-like peptide-1 (GLP-1) secretion in vitro Significantly stimulated GLP-1 secretion and was accompanied by enhanced insulin secretion in rats, which resulted in a reduced glycemic response after glucose injection	[25]
Genotypes raised in the National Agricultural Research Center for Kyushu Okinawa Region in Japan	Purification of 3,4,5-triCQA from sweet potato leaves	Caffeoylquinic acid derivatives	3,4,5-tri-O-caffeoylquinic acid	In vitro	100–500 µM	Inhibited aldose reductase Has the same IC_50_ value as epalrestat, a drug used in diabetic neuropathy	[26]
The sweet potato was grown in Kagawa Prefecture (Japan)	Powdered white sweet potatoes (Caiapo)	Protein Carbohydrate Fiber	-	In vivo	5 g/kg of BW/day for 4 weeks	Suppressed the increases of fasting plasma glucose and hemoglobin A1c levels Restored body weight loss during diabetes Increased serum insulin levels after oral glucose administration tests Reduced superoxide production from leukocytes and vascular homogenates, serum 8-oxo-2′ deoxyguanosine, and vascular nitrotyrosine formation of diabetic rats to comparable levels of normal control animals Depressed stress- and inflammation-related p38 mitogen activated protein kinase activity and tumor necrosis factor-α Improvement of pancreatic β-cells	[27]
White-skinned sweet potato	Powdered white sweet potatoes (Caiapo)	-	-	In vivo	Once daily 4 g for 5 months	Reduced HbA1c, fasting glucose and triglycerides Insulin remained unchanged. Increased adiponectin Decreased fibrinogen, no significant changes Improvement of insulin sensitivity	[28]
Fresh orange-fleshed (Jishu No. 16) sweet potato	Ethanolic fraction of distillated water extract	Trans-N-(p-coumaroyl)tyramine Trans-N-feruloyltyramine Cis-N-feruloyltyramine 3,4,5-tricaffeoylquinic acid 3,4-dicaffeoylquinic acid 3,5-dicaffeoylquinic acid 4,5-dicaffeoylquinic acid 4,5-feruloylcourmaoylquinic acid Caffeic acid Caffeic acid ethyl ester 7-hydroxy-5-methoxycoumarin Quercetin-3-O-α-D-glucopyranoside, 7,3′-dimethylquercetin Rhamnetin Indole-3-carboxaldehyde	Glucosidase inhibition: Trans-N-(p-coumaroyl)tyramine Trans-N-feruloyltyramine, Cis-N-feruloyltyramine 3,4,5-tricaffeoylquinic acid Caffeic acid ethyl ester 7-hydroxy-5-methoxycoumarin Indole-3-carboxaldehyde Antioxidant: 3,4,5-tricaffeoylquinic acid 3,4-dicaffeoylquinic acid 3,5-dicaffeoylquinic acid 4,5-dicaffeoylquinic acid 4,5feruloylcourmaoylquinic acid Caffeic acid Quercetin-3-O-α-D-glucopyranoside 7,3′-dimethylquercetin	In vitro	50 µL	α-glucosidase inhibition Antioxidant activity	[29]
The white-skinned sweet potato	Arabinogalactanprotein	arabinogalactanprotein	arabinogalactanprotein	In vivo	20 mg/kg BW of for 8 weeks	Reduced plasma glucose levels Changed insulin, TG, NEFA, leptin, and adiponectin levels Lowering Hs-CRP Suppressed the secretion of aggravating factors in insulin resistance Improvement in insulin sensitivity	[30]
White-skinned sweet potato	White-skinned sweet potato powder	Three fractions of WSSP (≤10, 10–50, and >50 kDa	-	In vivo	180–230 g/kg BW for 6–7 weeks	Reduced blood glucose levels Improvement of glucose tolerance Activated the phosphorylation of Akt, activating insulin signaling in the skeletal muscles and liver The ≤10 kDa fraction considerably reduced blood glucose levels per the OGTT and ITT. Suppressed gluconeogenesis and the expression of key enzymes in hepatocytes by the >50 kDa fraction Improved insulin sensitivity in skeletal muscles in normal rats	[31]
Caiapo^®^	A mixture of the pulverized tuber of Caiapo and the mulberry leaf Powder mixture of the pulverized skin of Caiapo and the powdered loquat leaf extract	-	-	In vivo	Caiapo (357 mg/kg BW) and the mulberry leaf powder (143 mg/kg BW) Pulverized skin of Caiapo (194 mg/kg BW) and the powdered loquat leaf extract (6 mg/kg BW)	Inhibited increase in blood glucose levels in the glucose loading test Lowering blood glucose levels at glucose tolerance test Reduction in blood glucose concentration	[32]
Purple sweet potato	96% ethanol (96%) and tartaric acid extract	-	-	In vivo	0.5 cc	Reduced high glucose levels in mice	[33]
White-skinned sweet potato	Lyophilized powder of distillated water tuber extract	-	-	In vivo	400 mg/kg BW/day	Lowered blood glucose Increased ACRP30 expression, which is a homolog of adiponectin Low tumor necrosis factor-a expression A greater tendency expression of the β-3-adrenoreceptor Improved action to the abnormal secretion of adipose tissue adipocytokines	[34]
Purple sweet potato	Commercial anthocyanin	Cyanidin-3- glucoside Cyanidin-3,5-glucoside Cyanidin-3-rutinoside Peonidin-3-glucoside	Cyanidin-3- glucoside Cyanidin-3,5-glucoside Cyanidin-3-rutinoside Peonidin-3-glucoside	In vitro and in silico	2.5, 5, 10, and 15 mg/mL	Inhibited porcine pancreatic α-amylase	[35]
White sweet potato (Simon No. 1)	Tuberous ethanol extract, ethyl-acetate and water fraction	The total phenolic content	-	In vitro	5–250 μg/mL	Increased the uptake of fluorescence glucose analogue (2-[N-(7-nitrobenz-2-oxa-1, 3-diazol-4-yl) amino]-2-deoxy-d-glucose, and 2-NBDG) in a dose-dependent manner Enhanced glucose uptake through activation of phosphorylation of IR (pIR), IRS-1 (pIRS-1) and Akt (pAkt) involved in PI3K (phosphatidylinositol 3-kinase)/protein kinase B (Akt) pathway, and up-regulated glucose transporter 4 (GLUT4) expression in myotubes.	[36]
Purple sweet potato powder cultivar Eshu No. 8	Anthocyanins	Diacylated and mono-acylated anthocyanins	Diacylated anthocyanins	In vitro and in vivo	160 mg/kg BW	Inhibited α-amylase and α-glucosidase Decreased blood glucose level	[37]
Orange-fleshed sweet potato cultivar ‘Bophelo’	Aqueous-methanol extracts of tuber (OSPT) and leave (OSPL)	Flavonoids Phenolic acid	Flavonoids Hyperoside Catechin Iso-orientin Kaempferol Orientin Quercetin Routine Vitexin Phenolic acid Caffeic acid isovanillic acid Protocatechuic acid Vanyllic acid	In vitro	500 μg/mL and 100 μg/mL of OSPT and OSPL	Increased intracellular GSH level, reduction in the level of malonaldehyde improvement in the intracellular antioxidant of the insulin resistant cells Modulated the expression levels of the type 2 diabetes-associated genes glucose transporter 4 (glut4), nuclear respiratory factor 1 (nrf1), myocyte enhanced factor 2A (mef2a), Carnitin palmitoy ltransferase 1 (cpt1), and Acetyl-CoA carboxylase 2 (acc2)	[38]
Korean purple sweet potato (ShinzamiSaeungbone9, Saeungyae33, Gyeyae2469, and Gyeyae2258)	15 individual anthocyanins	3-caffeoyl-p-hydroxybenzoyl-sophoroside-5-glucoside Peonidin 3-caffeoyl sophoroside-5-glucoside Peonidin 3-(6″-caffeoyl-6‴-feruloyl Sophoroside)-5-glucoside Peonidin 3-caffeoyl-p-hydroxybenzoyl-sophoroside-5-	Cyanidin 3-caffeoyl-phydroxybenzoylsophoroside-5-glucoside Peonidin 3-(6″-caffeoyl-6‴-feruloyl sophoroside)-5-glucoside	In vitro and in vivo	80 mg/kg BW	Antioxidant capacity (DPPH and ABTS) Inhibited glucose secretion in HepG2 cells (hepatic gluconeogenesis). Significantly lower blood glucose levels 1 and 2 h post-administration	[39]

## Data Availability

The data used to support the findings of this study can be made available by the corresponding author upon request.
